# Characterization of Cellular and Molecular Heterogeneity of Bone Marrow Stromal Cells

**DOI:** 10.1155/2016/9378081

**Published:** 2016-08-16

**Authors:** Mona Elsafadi, Muthurangan Manikandan, Muhammad Atteya, Jamil Amjad Hashmi, Zafar Iqbal, Abdullah Aldahmash, Musaad Alfayez, Moustapha Kassem, Amer Mahmood

**Affiliations:** ^1^Stem Cell Unit, Department of Anatomy, College of Medicine, King Saud University, Riyadh, Saudi Arabia; ^2^Molecular Endocrinology Laboratory (KMEB), Department of Endocrinology, University of Southern Denmark, Odense, Denmark; ^3^Department of Histology, Faculty of Medicine, Cairo University, Egypt; ^4^Center for Genetics and Inherited Diseases, Taibah University, Al-Madina Al-Munawara, Saudi Arabia; ^5^College of Applied Medical Sciences, King Saud Bin Abdulaziz University for Health Sciences (KSAU-HS), National Guards Health Affairs, Riyadh, Saudi Arabia; ^6^Prince Naïf Health and Research Center, College of Medicine, King Saud University, Riyadh, Saudi Arabia

## Abstract

Human bone marrow-derived stromal stem cells (hBMSC) exhibit multiple functions, including differentiation into skeletal cells (progenitor function), hematopoiesis support, and immune regulation (nonprogenitor function). We have previously demonstrated the presence of morphological and functional heterogeneity of hBMSC cultures. In the present study, we characterized in detail two hTERT-BMSC clonal cell populations termed here CL1 and CL2 that represent an opposing phenotype with respect to morphology, markers expression: alkaline phosphatase (ALP) and CD146, and* ex vivo* differentiation potential. CL1 differentiated readily to osteoblasts, adipocytes, and chondrocytes as shown by expression of lineage specific genes and proteins. Whole genome transcriptome profiling of CL1 versus CL2 revealed enrichment in CL1 of bone-, mineralization-, and skeletal muscle-related genes, for example,* ALP*,* POSTN*,* IGFBP5 BMP4*, and* CXCL12*. On the other hand, CL2 transcriptome was enriched in immune modulatory genes, for example,* CD14, CD99, NOTCH3, CXCL6, CFB*, and* CFI*. Furthermore, gene expression microarray analysis of osteoblast differentiated CL1 versus CL2 showed significant upregulation in CL1 of bone development and osteoblast differentiation genes which included several homeobox genes:* TBX15, HOXA2* and* HOXA10*, and* IGF1, FGFR3, BMP6, MCAM, ITGA10, IGFBP5*, and* ALP*. siRNA-based downregulation of the* ALP* gene in CL1 impaired osteoblastic and adipocytic differentiation. Our studies demonstrate the existence of molecular and functional heterogeneity in cultured hBMSC. ALP can be employed to identify osteoblastic and adipocytic progenitor cells in the heterogeneous hBMSC cultures.

## 1. Introduction 

Human bone marrow stromal (also known as skeletal or mesenchymal) stem cells (hBMSC) are increasingly employed in clinical trials for enhancing tissue regeneration following injury [1]. Typically, hBMSC are isolated by their ability to adhere to the plastic surfaces of* in vitro* culture plates. However, the cultured hBMSC exhibit morphological heterogeneity suggesting the presence of functional heterogeneity [2, 3]. It has also been suggested that the use of heterogeneous cell populations in clinical trials of hBMSC-based therapies caused variability in the observed treatment effects [4]. Thus, for the efficient use of hBMSC in therapy, better cellular and molecular characterization of hBMSC is required [1, 4].

There exist no specific markers that define the hBMSC phenotype. The plastic-adherent hBMSC are defined by the presence of surface expression of some CD surface markers with variable sensitivity and specificity [1]. Single cell clonal analysis revealed that only 25% of the cells are true stem cells based on their ability to differentiate into osteoblasts, adipocytes, and chondrocytes (trilineage differentiation) and to form heterotopic bone and bone marrow organ when implanted* in vivo* subcutaneously in immune deficient mice [5]. The identity of the remaining cells is not clarified, but they may represent lineage-committed cells [3]. Therefore, it is plausible that functional heterogeneity exists in cultured hBMSC, reflecting the* in vivo* functional and developmental heterogeneity of hBMSC [6].

In addition to their ability to differentiate into skeletal tissue cells (known as progenitor function), hBMSC possess immunomodulatory characteristics (known as nonprogenitor functions) [7]. It is not clear whether these different functions are mediated by a number of independent subpopulations within the hBMSC [2]. Only a few studies have tried to identify the subpopulation within cultured hBMSC based on surface markers, for example, STRO1 and alkaline phosphatase (ALP), but limited molecular phenotyping has been conducted [8].

We have previously demonstrated the presence of morphological and functional heterogeneity of clones isolated from telomerized hMSC (hMSC-TERT) cell line [3]. The aim of the present study was therefore to further study in detail the heterogeneity of cultured hBMSC as demonstrated by two clonal cell lines with opposite cellular and functional phenotype. We also employed the DNA microarrays to define their molecular signature and signaling pathways associated with their functional phenotype.

## 2. Experimental Procedures

### 2.1. Cell Culture

As a model for hBMSC, we employed immortalized hBMSC-TERT cell line that is created from normal human BMSC by overexpression of human telomerase reverse transcriptase gene (hTERT) [9]. The hBMSC-TERT cells have been extensively characterized, and they exhibit similar cellular and molecular phenotype to primary MSC [10]. CL1 and CL2 cells are clonal cell populations of hBMSC-TERT identified in long term culture (passage numbers 15–25) of hBMSC-TERT and were chosen based on their distinct and different morphologies. Cells were cultured in Dulbecco's Modified Eagle Medium (DMEM) supplemented with D-glucose 4500 mg/L, 4 mM L-glutamine and 110 mg/L sodium pyruvate, 10% Fetal Bovine Serum (FBS), 1x penicillin-streptomycin (Pen-strep), and nonessential amino acids (all purchased from Gibco-Invitrogen, USA). For some control experiments, primary bone marrow derived MSC (phBMSC) were employed. Sixty milliliters of bone marrow was aspirated from the iliac crest bone of consenting healthy donors. This procedure was approved by the King Khalid University Hospital-King Saud University ethics committee. phBMSC were isolated from bone marrow mononuclear cells by plastic adherence as described previously [9].

### 2.2. Cell Proliferation

Cell proliferation rate was determined by counting cell number and calculating population doubling (PD) rate. The cells were cultured in 25 cm^2^ tissue culture Petri dish at cell density 0.5 × 10^6^ cells (28000 cells/cm^2^). At confluence, the cells were trypsinized and counted manually by hemocytometer. At each passage, population doubling was determined by the following formula: log⁡*N*/log⁡2, where *N* is the number of cells at confluence divided by the initial cell number. Cumulative PD level is the sum of population doublings, and PD rate is PD/time in culture.

### 2.3. Flow Cytometry

Cells were trypsinized to a single cell suspension, were recovered by centrifugation at 200 g for 5 min, washed twice in ice-cold PBS supplemented with 2% BSA, and resuspended at a concentration of 10^5^ cells/antibody. After incubation with the preconjugated antibodies, or matched isotype controls, for 30 min on ice in the dark, cells were washed with PBS, resuspended in 500 *μ*L of PBS, and analyzed in the BD FACSCalibur flow cytometer (BD Biosciences). Living cells were gated in a dot plot of forward versus side scatter signals acquired on a linear scale. At least 10,000 gated events were acquired on a log fluorescence scale. Positive staining was distinct as the emission of a fluorescence signal that surpassed levels achieved by >99% of control cell population stained with corresponding isotype antibodies. The ratios of fluorescence signals versus scatter signals were calculated, and histograms were generated using the software Cell Quest Pro Software Version 3.3 (BD Biosciences). The following antibodies were used all from BD Biosciences: FITC-PE-APC-Mouse IgG1k isotype control, APC-Mouse Anti-Human CD44 (#559942), FITC-Mouse Anti-Human CD63 (#557305), PE-Mouse Anti-Human CD73 (#550257), PE-Mouse Anti-Human CD105 (#560839), PE-Mouse Anti-Human CD146 (#550315), PE-Mouse Anti-Human CD166 (#560903), and Alexa Fluor® 488 Mouse Anti-Human Alkaline Phosphatase (#561495).

### 2.4. Electron Microscopy

After trypsinizing the hBMSC cells from the flasks or 6-well plates were collected, the samples were washed with PBS, and the pellets were resuspended directly in 2.5% glutaraldehyde in 0.1 M phosphate buffer (pH 7.2), and kept at 4°C for 4 hr. First, the cells were washed with 0.1 M phosphate buffer (pH 7.2) and transferred to 1% osmium tetroxide (OsO4) solution in 0.1 M phosphate buffer (pH 7.2) for two hr. The cells were dehydrated in ascending grades of ethanol. The cells were then resuspended in acetone and were aliquoted into BEEM embedding capsules and infiltrated with acetone: resin mixture followed by embedding in a pure resin mixture for two hr. Semithin sections (0.5 *μ*m thickness) and ultrathin sections (70 nm thickness) were prepared, examined, and photographed under a transmission electron microscope (TEM) (Jeol 1010, Jeol, Tokyo, Japan).

### 2.5. *In Vitro* Osteoblast Differentiation

Cells were grown in standard DMEM growth medium in 6-well plates at 20,000 cell/cm^2^. When 70–80% confluence was achieved, test cells were cultured in DMEM supplemented with osteoblastic induction mixture (referred to as OS) containing 10% FBS, 1% Pen-strep, 50 *μ*g/mL L-ascorbic acid (Wako Chemicals, Neuss, Germany), 10 mM *β*-glycerophosphate (Sigma) and 10 nM calcitriol (1*α*,25-dihydroxyvitamin D3; Sigma), and 10 nM dexamethasone (Sigma); noninduced cells (referred to as Cont) were cultured in normal growth media for the same duration as induced. The media were replaced three times per week. Cells cultured in standard culture medium were considered as control. At day 14 of differentiation, mineralized nodules became apparent and were stained with Alizarin Red S and ALP.

### 2.6. *In Vitro* Adipocyte Differentiation

Cells were grown in standard DMEM growth medium in 6-well plates at 0.3 × 10^6^ cells/mL. At 90–100% confluence, cells were cultured in DMEM supplemented with adipogenic (Adip) induction mixture containing 10% FBS, 10% Horse Serum (Sigma), 1% Pen-strep, 100 nM dexamethasone, 0.45 mM isobutyl methylxanthine (Sigma), 3 *μ*g/mL insulin (Sigma), and 1 *μ*M Rosiglitazone (Novo Nordisk, Bagsvaerd, Denmark). The media were replaced three times per week. Cells cultured in standard culture medium were considered as control. From day 3 of differentiation, small lipid droplets became visible and at day 7 they were stained with Oil Red-O and Nile red.

### 2.7. *In Vitro* Chondrogenic Differentiation

Both CL1 and CL2 cells were trypsinized and counted, around 1 × 10^6^ cells taken in each 15 mL conical tube centrifuged at 400 ×g for 5 minutes. For chondrocyte differentiation pellet culture system used, chondrocyte induction was done in media containing advanced DMEM/F12 supplemented with 1% ITS Premix Tissue Culture Supplement, 100 nM dexamethasone, Glutamax, and 10 ng/mL transforming growth factor-beta-3 (TGF*β*-3). Cells were maintained in chondrocyte differentiation media for 21 days and changed every two days.

### 2.8. Cytochemical Staining

#### 2.8.1. Alkaline Phosphatase (ALP) Staining

CL1 and CL2 cells were stained before OS differentiation for the basal ALP expression and after OB differentiation at day 7 of induction. Cells cultured in 6-well plates were washed in PBS −/− (-Ca, -Mg) and fixed in acetone/citrate buffer 10 mM at pH 4.2 for 5 min at room temperature. The Naphthol/Fast Red stain [0.2 mg/mL Naphthol AS-TR phosphate substrate (Sigma)] [0.417 mg/mL of Fast Red (Sigma)] was added for one hour at room temperature.

Histological tissue blocks were sectioned at 4 microns. Immunohistochemical staining was performed on CL1 and CL2 chondrocyte 3D pellets using DAKO EnVision and PowerVision according to the manufacturer's instructions (DAKO, Glostrup, Denmark). Briefly, paraffin sections were incubated for 1 hour at room temperature with primary antibodies diluted in ChemMate (DAKO) (Human Anti-Col-10 and Human Anti-Col-2 ABI). Sections were washed subsequently in Tris-buffered saline (TBS, 0.05 M, pH 7.4), incubated for 30 minutes with secondary anti-mouse Ig/HRP-conjugated polymers (K4001, En Visionþ, DAKO), and visualized with 3,30-diaminobenzidine tetrahydrochloride (DAB, S3000, DAKO) or with 3-amino-9-ethylcarbazole (AEC, DAKO) according to manufacturer's instruction. Controls were performed with nonimmune immunoglobulins of the same isotype as the primary antibodies (negative controls) and processed under identical conditions. Alcian blue staining was used to detect chondrocytes. Sections of paraffin-embedded implants were stained with Alcian blue (Sigma) solution, pH 2.5; at this pH all the glycoproteins (neutral and acidic) will be stained blue.

### 2.9. Alizarin Red S Staining for Mineralized Matrix

Seven-day-old OS differentiated cells in 6-well plates were used for Alizarin Red S staining. The cell layer was washed with PBS and then fixed with 70% ice-cold ethanol for 1 hr at −20°C. After removing the ethanol, the cell layer was rinsed with distilled water and stained with 40 nM AR-S (Sigma) pH 4.2 for 10 minutes at room temperature. Excess dye was washed off with water followed by a wash with PBS for few minutes to minimize nonspecific AR-S stain.

For quantifying the Alizarin Red S staining, the air-dried plates, the Alizarin Red S dye was eluted in 800 *μ*L of acetic acid incubated in each well for 30 minutes at room temperature as described [11] and measured in spectrophotometer (BioTek, Epoch) at 405 nm.

### 2.10. Quantitative ALP Activity

To quantify ALP activity in CL1 and CL2 hBMSC before and after OS differentiation, we used the BioVision ALP activity colorimetric assay kit (BioVision, Inc, CA, USA) with some modifications. Cells were cultured in 96-well plates under normal conditions; then on day of analysis, wells were rinsed once with PBS and were fixed using 3.7% formaldehyde in 90% ethanol for 30 seconds at room temperature. Subsequently, fixative was removed, and 50 *μ*L of pNPP solution was added to each well and incubated for 1 hour in the dark at room temperature. The reaction was subsequently stopped by adding 20 *μ*L stop solution and gently shaking the plate. OD was then measured at 405 nm.

### 2.11. Oil Red-O Staining for Lipid Droplets

CL1 and CL2 cells differentiated to adipocytes with Adip induction media at day 7 were used. Accumulated cytoplasmic lipid droplets were visualized by staining with Oil Red-O. After washing cells grown in 6-well plates with PBS, the cells were fixed in 4% formaldehyde for 10 min at room temperature and then rinsed once with 3% isopropanol and stained for 1 hr at room temperature with filtered Oil Red-O staining solution (prepared by dissolving 0.5 g Oil Red-O powder in 60% isopropanol). To quantify staining of fat droplets, Oil Red-O was used as a stain. Oil Red-O was eluted by adding 100% isopropanol to each well, and color changes were measured by spectrophotometer at 510 nm (BioTek Spectrophotometer, Epoch).

### 2.12. Nile Red Fluorescence Determination and Quantification of Adipogenesis

A stock solution of Nile red (1 mg/mL) in DMSO was prepared and stored at −20°C protected from light. Staining was performed on unfixed cells. Cultured undifferentiated and day 7 adipocyte differentiated cells were grown in Corning polystyrene; flat bottom 96-well TC-treated black microplates (Corning, NY, USA) were washed once with PBS. The dye was then added directly to the cells (5 *μ*g/mL in PBS), and the preparation was incubated for 10 min at room temperature and then washed twice with PBS. Fluorescent signal was measured using SpectraMax/M5 fluorescence spectrophotometer plate reader (Molecular Devices Co, Sunnyvale, CA, USA) using bottom well-scan mode where nine readings were taken per well using Ex (485 nm) and Em (572 nm) spectra.

### 2.13. Quantitative Real-Time PCR (qRT-PCR) Analysis

Total RNA was extracted using MagNA pure compact RNA isolation kit (Roche Applied Science, Germany, Cat number 04802993001) in an automated MagNA pure compact system (Roche, Germany) as recommended by the manufacturer. The total RNA was quantified by Nanodrop spectrophotometer (Nanodrop 2000, Thermo Scientific, USA). Complementary DNA (cDNA) was synthesized from 1 *μ*g of the RNA samples using High Capacity cDNA Reverse Transcription kit (Applied Biosystems, USA) using Labnet, Multigene thermocycler according to the manufacturer's instructions. Relative levels of mRNA were determined from cDNA by real-time PCR (Applied Biosystems-Real-Time PCR Detection System) with Power SYBR Green PCR kit (Applied Biosystems, UK) according to the manufacturer's instructions. Following normalization to the reference gene GAPDH, quantification of gene expression was carried out using a comparative Ct method, where ΔCt is the difference between the CT values of the target and the reference gene, and fold induction was performed from the control (Cont) for the same time point. Primers (Supplementary Table  1 in Supplementary Material available online at http://dx.doi.org/10.1155/2016/9378081) were obtained from Applied Biosystems (USA) as TAQMAN primers, or previously published primers were used (see Supplementary Table  1).

### 2.14. DNA Microarray Global Gene Expression Analysis

Four hundred ng of total RNA was used as input for generating biotin-labeled cRNA (Ambion, Austin, TX, United States). cRNA samples were then hybridized onto Illumina® human-8 BeadChips version 3. Hybridization, washing, Cy3-streptavidin staining, and scanning were performed on the Illumina BeadStation 500 platform (Illumina, San Diego, CA, USA), according to the manufacturer's instructions, and everything was done in triplicate. Expression data analysis was carried out using the Partek® genomic suite software. Raw data were background-subtracted, normalized using the “rank invariant” algorithm, and filtered for significant expression on the basis of negative control beads. Genes were considered significantly expressed with detection *p* values ≤ 0.01. Differential expression analysis was performed with the Illumina custom method using freshly isolated primary hBMSC (used at passage 3) as a reference control. The following parameters were set to identify statistical significance: differential *p* values ≤ 0.01; fold change ratio > 1.5. Pathway analysis was performed using DAVID Bioinformatics Resources 6.7 (http://david.abcc.ncifcrf.gov/) and GeneSpring GX software (Agilent Technologies). Pathway analysis for CL1 OS D14 versus CL2 OS D14 was conducted using the Single Experiment Pathway analysis feature in GeneSpring 12.0 (Agilent Technologies).

### 2.15. Small Interfering (si)RNA Transfection

For transfection, hBMSC in logarithmic growth phase were transfected with Silencer Select Predesigned ALP siRNA (25 nM) (Assay ID; s1298 and Cat number 4390824) (Ambion, The RNA Company, USA) using Lipofectamine RNAiMAX Reagent (Invitrogen, CA, USA) plus serum-free Opti-MEM®I medium under the conditions described by the manufacturer. At day 3 of transfection, the cells were induced for osteogenic differentiation for an additional 7 days. ALP staining was used as a control for the siRNA transfection efficiency and timeline.

### 2.16. Statistical Analysis

All of the results were presented as the mean and standard deviation (SD) of at least 3 independent experiments, with 3–5 technical repeats in each experiment. Student's *t*-test (two-tailed) was used for testing differences between groups. *p* value <0.05 was considered statistically significant.

## 3. Results

### 3.1. Comparison between CL1 and CL2: Differences in Morphology, Proliferation, and Marker Expression Profile

**Figure 1:**
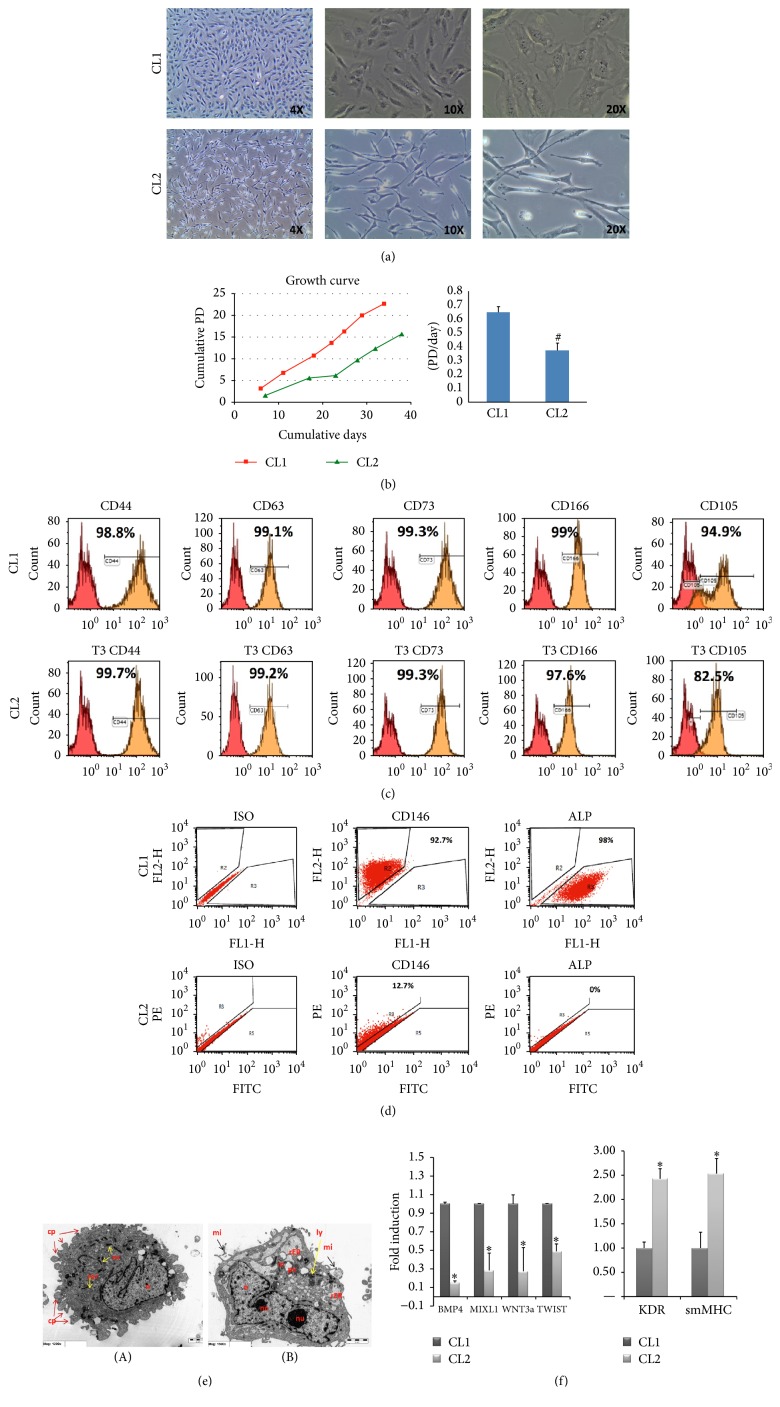
Cellular phenotype of two clonal populations of human bone marrow stromal stem cells: CL1 and CL2. (a) Cell morphology. CL1 cells showed large cuboidal morphology while CL2 cells had spindle-shaped fibroblast-like morphology. (b) Growth curves showing population doubling (PD) rate during long term culture. (c) Flow cytometry analysis (FACS) shows expression of CD44, CD63, CD73, CD105, and CD166 in CL1 and CL2 cells. Matched isotype control was used for gating. (d) Flow cytometry analysis presented as dot blot of CD146 and alkaline phosphatase (ALP) cell surface proteins. (e) Transmission electron microscope (TEM). (A): CL1 (1200x); (B): CL2 (1500x). n: nucleus, nu: nucleolus, rER: rough endoplasmic reticulum, ly: lysosomes, pv: phagocytic vacuole, and rer: reticular stalk of rER. (f) Gene expression analysis using RT-PCR for a group of mesodermal and stromal genes. Gene expression was normalized to GAPDH and presented as fold change. Data is shown as mean ± SD of three independent experiments. ^*∗*^ *p* < 0.05; ^#^ *p* < 0.001.

We isolated two distinct clonal cell populations of hBMSC-TERT: hBMSC-CL1 and hBMSC-CL2 (for easiness will be termed hereafter CL1 and CL2) based on differences in cell morphology (Figure 1(a)). CL1 cells had cuboidal morphology whereas CL2 cells have spindle-shaped fibroblast-like morphology. CL1 cells had higher proliferation rate compared to CL2 (Figure 1(b)): mean PD rates of CL1 and CL2 were 0.714 and 0.429 PD/day, respectively (Figure 1(b)). Both CL1 and CL2 expressed surface marker profiles characteristics of hBMSC (>90%): CD44+, CD63+, CD73+, CD105+, and CD166+ (Figure 1(c)). However CL1 cells showed higher expression of CD146 (92.7% versus 12%) and ALP (98% versus 0%) compared to CL2 (Figure 1(d)). TEM revealed the presence of abundant pseudopodia in CL1 indicating high motility (Figure 1(e)(A)) as well as well-developed mitochondria and rough endoplasmic reticulum (rER) suggesting high metabolic activity. CL2 cells contained abundant phagocytic vacuole (pv), microvilli (mi), and lysosomes (ly) (Figure 1(e)).

We performed quantitative real-time PCR (RT-PCR) for genes expressed in mesodermal progenitor cells [12]. CL1 expressed higher levels of BMP4, MIXL1, WNT3a, and TWIST compared to CL2 (Figure 1(f), *p* < 0.01). In contrast, CL2 expressed higher levels of Kinase Insert Domain Receptor (Type III Receptor Tyrosine Kinase) (KDR) expressed in endothelial cells and smooth muscle myosin heavy chain gene (smMHC) expressed in smooth muscle cells (Figure 1(f)).

### 3.2. CL1 Cells Exhibit Enhanced Osteoblast Differentiation

**Figure 2:**
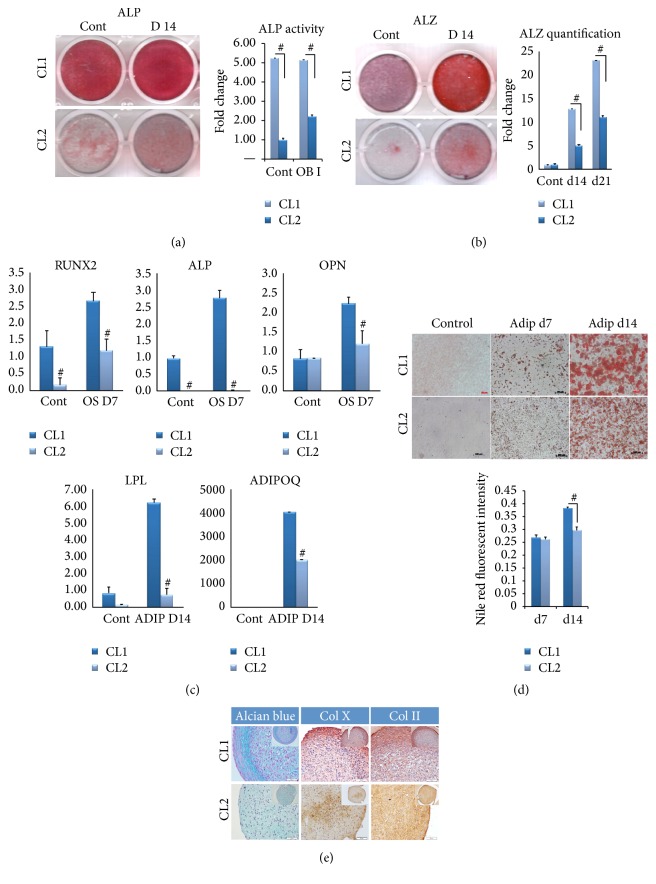
*In vitro* osteoblastic and adipocytic differentiation of two clonal populations of human bone marrow stromal stem cells: CL1 and CL2 cells. Both cell lines were induced for osteoblast differentiation using standard protocol described in the Methods. (a) ALP staining at day 14 in control noninduced (Cont) and osteoblast induced cells (D14). Right panel shows ALP activity (*n* = 3 independent experiments, ^#^ *p* < 0.001). (b) Mineralized matrix formation visualized by Alizarin Red S staining. Right panel shows Alizarin Red quantification at day 14 and 21 after osteoblast differentiation (*n* = 3 independent experiments, ^#^ *p* < 0.001). (c) Quantitative RT-PCR of osteoblastic and adipocyte gene markers in CL1 and CL2 during osteoblast (upper panel) and adipocyte (lower panel) differentiation. ALP = alkaline phosphatase, OPN = osteopontin, LPL = lipoprotein lipase, and ADIPOQ = adiponectin. Data are presented as fold change in expression of each target gene normalized to GAPDH (*n* = 3 independent experiments, *p* < 0.05;  ^#^ *p* < 0.001). (d) CL1 and CL2 lines were induced for adipocyte differentiation using standard protocol described in the Methods. Adipocyte formation was visualized at day 7 (Adip d7) and day 14 (Adip d14) by Oil Red-O staining. Lower panel presents quantification of Nile red staining (*n* = 3 independent experiments, ^#^ *p* < 0.001). (e) CL1 and CL2 lines were induced for chondrocyte differentiation using 21-day pellet culture method as described in the Methods. The pellets were stained with Alcian blue, collagen 10 (Col X), and collagen 2 (Col II) (original magnification 5x).

Following osteoblast (OB) differentiation induction, ALP staining and ALP enzymatic activity were significantly higher in CL1 compared to CL2 cells (Figure 2(a), *p* < 0.01). Similarly, Alizarin Red staining and quantitation of formed mineralized matrix were more pronounced in CL1 cells (Figure 2(b), *p* < 0.01). In addition, CL1 cells expressed higher levels of osteoblastic genes, ALP, RUNX2, and osteopontin (OPN) (Figure 2(c) upper panel) compared to CL2 cells.

Global gene expression microarray analysis of OB differentiated cells at day 14 showed around 1060 genes significantly upregulated more than 2-fold (*p* < 0.01) in CL1. Among the upregulated genes, 80 genes were annotated to bone development and osteoblast differentiation (Table 1). The highest upregulated genes included paired-like homeodomain 2 (PITX2), Insulin-like growth factor 1 (IGF1) and collagen, type V, alpha 3 (COL5A3), osteomodulin (OMD), and T-box 15 (TBX15) (Table 1). Furthermore, several known osteoblast-related genes were upregulated in CL1 cells such as bone morphogenetic protein 6 (BMP6), fibroblast growth factor receptor 3 (FGFR3), insulin-like growth factor binding protein 5 (IGFBP5), and vitamin D (1,25-dihydroxyvitamin D3) receptor (VDR) (Table 1). On the other hand, 1200 genes were upregulated in CL2 cells: 255 genes were annotated to immunity and immune response and defense. This category included genes from, complement system, chemokine (C-C motif) ligands, interferon family, chemokine (C-X-C motif) ligands, and receptor, major histocompatibility complex class II molecules, interleukins, and tumor necrosis factor receptor superfamily (Table 2 and Supplementary Table 3).

### 3.3. CL1 Cells Exhibit Enhanced Adipocyte Differentiation

We observed significant differences between CL1 and CL2 in their response to adipocytic differentiation induction. CL1 differentiated readily to adipocytes compared to CL2 (Figure 2(d), lower panel) evidenced by higher levels of adipocytic markers gene expression, LPL (lipoprotein lipase), and adiponectin, as well as formation of mature lipid filled adipocytes visualized by Oil Red-O staining and quantitative Nile red staining (Figure 2(d)).

### 3.4. CL1 Cells Differentiate to Chondrocytic Lineage

In pellet cultures, CL1 cells formed 3D pellets containing proteoglycan-secreting chondrocytes, which stained positive with Alcian blue. Limited chondrocyte differentiation was visible in cell pellets of CL2 cells. The differentiated chondrocytes in CL1 pellets expressed higher levels of collagen X and collagen II, which was overlapping the Alcian blue stain (Figure 2(e)).

### 3.5. Molecular Signature of CL1 and CL2 Cells

**Figure 3:**
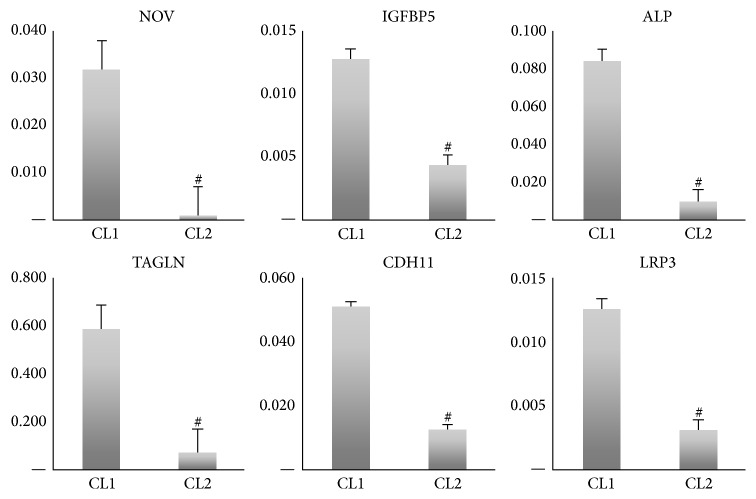
Validation of whole genome microarray analysis of two clonal populations of human bone marrow stromal stem cells: CL1 and CL2 cells. Quantitative real-time PCR for highly expressed genes in CL1 cells. NOV = nephroblastoma overexpressed, IGFBP5 = insulin-like growth factor binding protein 5, ALP, TAGLN = transgelin, and CDH11 = OB-cadherin (osteoblast). Data are presented as fold change in expression of each target gene normalized to GAPDH (*n* = three independent experiments, ^#^ *p* < 0.001) (see also Table 3).

To define the molecular signature and molecular differences between CL1 and CL2, we compared the basal gene expression pattern of CL1 and CL2 cells using DNA microarrays. The PCA analysis showed a clear separation between CL1 and CL2 (Supplementary Figure 1). Comparison between CL1 with CL2 showed that 915 genes were differentially expressed in the two cell lines (>2-fold, *p* < 0.01): 462 genes were upregulated, and 452 were downregulated in CL1 versus CL2. The most relevant genes that were upregulated in CL1 are listed in Table 3(a). Among these 35 highly expressed genes in CL1, the following 11 genes were present in skeletal and muscular system development and function: FOLR3, CCL3L1, SERPINB2, POSTN, IGFBP5, CCL3, NOV, ALP, TNFRSF11B, ACTG2, and CDH11 (Table 3(a)). Functional annotation of the upregulated genes in CL1 using the Ingenuity Pathway Analysis (IPA) revealed enrichment in the following categories: “tissue development,” “skeletal and muscular system development and function,” and “organismal development” (Table 3(b)). Furthermore, the DAVID annotation tool was employed to assess the functional relationships of the upregulated genes in CL1 showing enrichment in ontologies: “skeletal and muscular system development and function” that included bone size, osteoblast differentiation, bone mineralization, and bone mineral density (Table 3(c)). CL1 exhibited upregulation of WNT pathway ligands: WNT5B (2-fold) and LRP5 (2-fold) (Table 1). Also, ALP was among the highly expressed genes together with POSTN, IGFBP5, SPP1, IL-6, and DKK1 (Tables 3(a) and 1). These genes are known to play an important role in osteoblast differentiation and bone formation. For CL2, inhibitors of WNT pathway were upregulated and included SFRP1 (11-fold), DKK2 (3.2-fold), FGF2 (3.1-fold), and GBP2 (2.4-fold). Functional annotation of the upregulated genes in CL2 revealed enrichment in the following categories: “developmental process,” “multicellular organismal process,” “biological adhesion,” and “immune system process” (Supplementary Table 2A). In-depth analysis of the biological processes revealed several immune-related pathways: “MAPKKK cascade,” “immunity and defense,” “signal transduction,” “extracellular matrix protein-mediated signaling,” and “interferon-mediated immunity,” among others that were upregulated (Supplementary Table 2B). Also, 40 genes related to immune system related factors were identified as significantly enriched in CL2 compared to CL1 cells (Supplementary Table 2C). We chose the following genes for validation of the microarray results: NOV, IGFBP5, ALP, TAGLN, and CDH11 as they were highly expressed in CL1. RT-PCR analysis confirmed the microarray results (Figure 3).

Furthermore, we compared the molecular phenotype of CL1 and CL2 cells with that of phBMSC. We found that more than 80% of the genes expressed in CL1 and 90% in CL2 cells were common with primary phBMSC (Supplementary Figure 2), suggesting that CL1 and CL2 molecular phenotype exist within the heterogeneous population of phBMSC cultures.

### 3.6. ALP Knockdown Impairs Differentiation of CL1 Cells

**Figure 4:**
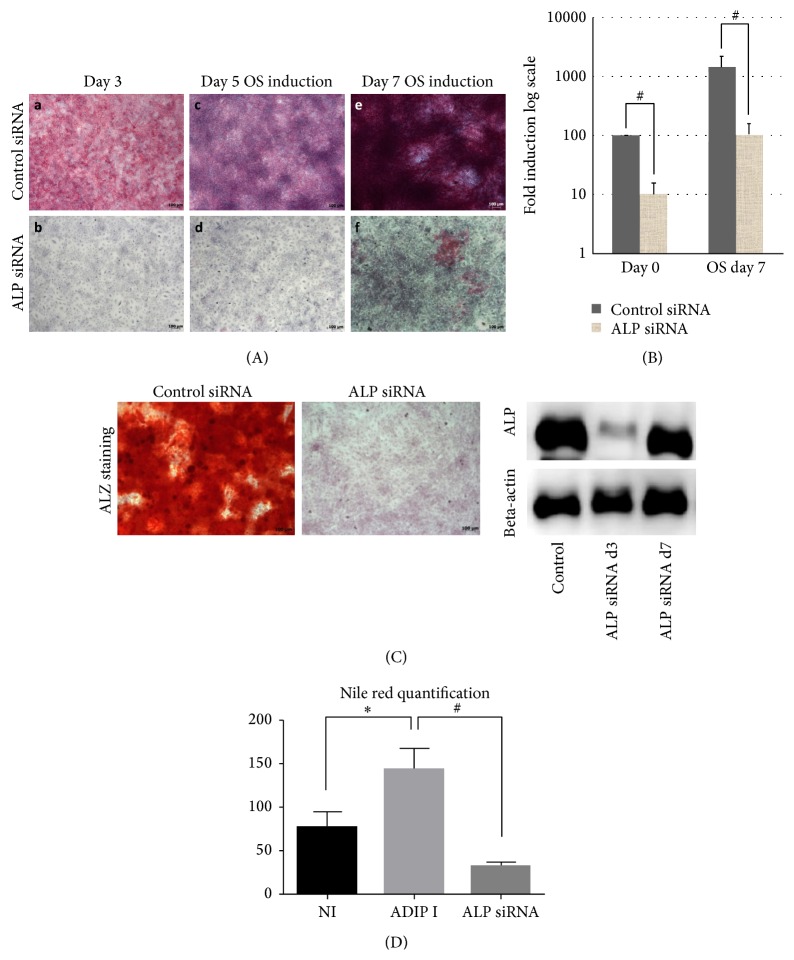
Effect of alkaline phosphatase (ALP) gene silencing by small interfering RNA (siRNA) on a clonal population of human bone marrow stromal stem cell CL1. (A) ALP staining at day 3, day 5, or day 7 days of osteoblast differentiation (OB induction). (B) Quantitative real-time PCR for ALP gene following ALP siRNA transfection at day 0 OB and day 7 of OB. Data are presented as fold change in expression of each target gene normalized to GAPDH (*n* = three independent experiments, ^#^ *p* < 0.01). Western blotting analysis of day 3 and day 7 after siRNA ALP transfection of CL1 cells, ALPL specific antibody, and B-actin was used. (C) Mineralized matrix formation as visualized by Alizarin Red S staining in siRNA transfected CL1 cells after 14 days of OB induction. (D) Nile red quantification of mature lipid filled adipocyte in control noninduced (Cont), adipocyte induced (Adip I), and ALP siRNA transfected cells that are adipocyte induced (ALP siRNA). Adipocyte induction was carried out for 7 days. ^*∗*^ *p* < 0.05.

Since ALP has been suggested as a marker for hBMSC progenitor cell lineage commitment [13] and was highly upregulated in CL1 cells, we tested its biological role in CL1 cells. ALP siRNA transfection decreased ALP protein level, ALP activity, and mRNA gene expression compared to control cells transfected with control siRNA (*p* < 0.01) and this inhibition was detectable up to day 7 days after osteoblast differentiation induction (Figures 4(A) and 4(B)). At day 14 of differentiation, mineralization ability of CL1 was significantly impaired (Figure 4(C)). In addition, we found that the number of mature adipocyte formations was significantly reduced to more than 75% (*p* < 0.01) (Figure 4(D)).

**Figure 5:**
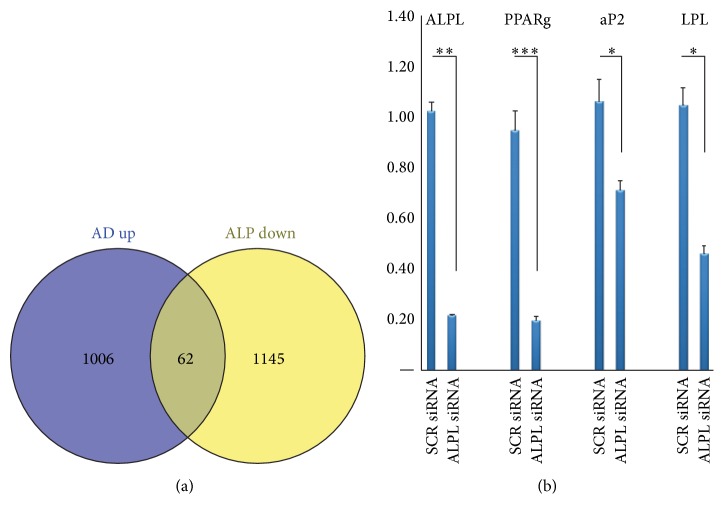
Adipocyte related genes downregulated in ALP knockdown CL1 cells. (a) Venn diagram of whole gene expression analysis of AD upregulated genes compared with ALP KD downregulated genes. (b) Quantitative real-time PCR of four selected common genes from Venn diagram including ALPL, PPARg2, aP2, and LPL. Data are presented as fold change in expression of each target gene normalized to GAPDH (*n* = three independent experiments, ^*∗*^ *p* < 0.05, ^*∗∗*^ *p* < 0.01, and ^*∗∗∗*^ *p* < 0.001).

To identify relevant adipocyte differentiation associated genes that were targeted by ALP deficiency, we compared the downregulated genes of ALP deficient CL1 with the upregulated genes identified during adipocytic differentiation of CL1. We identified 62 genes that were common (Figure 5(a), Table 4) and among these genes were genes related to metabolism (primarily lipid and carbohydrate) and transport including CYB5B, CHST1, TAP1, ATP8A1, LRP8, PLCD1, and FABP5 (Table 4). We further performed quantitative real-time PCR of ALP deficient CL1 cells during adipocyte differentiation. The following adipocyte-associated genes were downregulated: PPAR*γ*2, LPL, and aP2 (Figure 5(b)), confirming impairment of adipocytic differentiation of ALP deficient CL1 cells.

## 4. Discussion 

We extensively studied two cell populations within cultured hBMSC that were identified based on differences in morphology. Cellular and molecular studies revealed differences in growth, differentiation capacity, and molecular signature. Our data support the notion of the presence of cellular and functional heterogeneity among cultured hBMSC.

Cellular heterogeneity of cultured hBMSC is recognized in an increasing number of reports. Several extrinsic and intrinsic factors may contribute to the observed hBMSC heterogeneity. Extrinsic factors include donor-to-donor variations in the number and quantity of initiating cells, which result in differences in cell growth rate and differentiation capacity [14, 15]. Intrinsic factors have been examined employing single cell clonal analysis and revealed variations in differentiation potential among individual colonies within hBMSC cultures ranging from the presence of cells with trilineage (osteoblast, adipocytes, and chondrocyte) potency to cells with null potency [16]. Also, variations in the ability of clonal cells to form heterotopic bone when implanted* in vivo* have been reported [5]. Our study corroborates these findings and provides more detailed cellular and molecular phenotyping of two examples of cell populations that exist within the heterogeneous hBMSC cultures [17].

Determining the molecular signature of CL1 and CL2 using whole genome microarray analysis showed enrichment of lineage-commitment associated genes in CL1. For example, insulin-like growth factor 5 (IGFBP5) and interleukin 6 (IL6) were 14.7- and 3.3-fold upregulated in CL1 cells, respectively. Both factors are expressed in osteoprogenitor cells and important for osteoblast maturation [18]. We also observed that periostin (POSTN) gene was highly upregulated in CL1 cells (15.6-fold); POSTN is a 90 kDa secreted protein, originally identified in murine osteoblast-like cells and is upregulated by PTH [19]. Several studies employing murine and human cells have revealed important role of POSTN in osteoblast differentiation and during development in intramembranous ossification [20–23]. Another factor identified in CL1 cells is nephroblastoma overexpression (NOV) which is a member of the Cyr 61, connective tissue growth factor (CNN) family. The CCN family of proteins promotes osteoblast differentiation through interaction with integrins, WNT, BMP, and NOTCH signaling pathways [24–26]. In addition, a large number of signaling molecules known to be regulators of hBMSC lineage specific differentiation, for example, insulin-like growth factors [27–29], WNT [30–32], and MAPK [33–35], were enriched in CL1 cells. In contrast, CL2 expressed high levels of immune-related genes which may explain the poor differentiation response to osteoblast or adipocyte lineage. In a recent study the authors used telomerized hBMSC and showed clearly a clonal population that had very low* in vitro* and* in vivo* differentiation ability; however they had enhanced immune-related features including high IL7 expression. These nullipotent cells expressed CD317 which was associated with remarkably high basal level expression of factors with a proinflammatory and antiviral function [17]. We observed that this molecular phenotype was associated with distinct ultrastructural characteristics of the cells. In particular, CL2 had abundant phagocytic vacuole, microvilli, and lysosomes, features reminiscent of ultrastructure of immune-regulatory cells. Our data thus support the increasingly recognized feature that hBMSC exhibit immune modulatory functions and a part of the innate immune response [17].

We observed that ALP protein expression and enzymatic activity were significantly different between CL1 and CL2 cell lines and were thus a potential marker that distinguishes different cell populations with progenitor functions (CL1) from cells with nonprogenitor functions (CL2). ALP is expressed in a wide variety of tissues, including kidneys, bone, and liver [36, 37], but tissue-nonspecific ALP (ALPL) is considered a commitment marker for osteoblastic lineage [13, 38]. However, in a recent study the authors examined the differentiation potential of a number of hMSC clones* in vitro* and* in vivo* and reported that the hMSC clones with high levels of ALP expression were committed to trilineage differentiation [13]. Our data corroborate and extend these findings by reporting the effects of siRNA-mediated inhibition of ALP that resulted in an impaired hBMSC differentiation not only to osteoblasts, but also to adipocytes. Also, our results corroborate earlier studies that demonstrated in human bone biopsies the presence of ALP expression in bone marrow adipocytic cells [13, 17]. All these data suggest that ALP is a “stemness” marker of hBMSC and not just an indicator of osteoblastic lineage commitment.

While CL1 and CL2 were isolated from telomerized hMSC cell line, they are relevant to normal human physiology. We observed that the molecular phenotypes of CL1 and CL2 were contained within the molecular signature of primary hBMSC suggesting that CL1 and CL2 represent cell populations within the heterogeneous cultures of hBMSC. We have also previously reported that the molecular phenotype and cellular responses of hMSC-TERT are similar to those of primary hMSC [10]. While we have identified ALP as a marker that can be used for a prospective identification of differentiation committed population of hBMSC, we identified additional distinctive molecular markers of the cells. For example, IGF-1, IGF-2, and IGF binding protein 5 were enriched in CL1 compared to CL2. IGFs and their binding proteins are very well-studied factors that play a role in hBMSC proliferation and osteoblast differentiation [18]. On the other hand, annexin A3 as well as several immune-related genes was highly enriched in CL2 compared to CL1. Future studies are needed to determine the functional significance of these molecules in relation to the functional identity of various cell populations within the hBMSC cultures and their usefulness as biomarkers to dissect the heterogeneous population of cultured hBMSC.

Our finding of the presence of functional diversity within hBMSC cultures that contain progenitor and nonprogenitor cell populations has a clinical relevance. It demonstrates that the progenitor function and the immune modulatory roles of hBMSC [39] are mediated by specific and distinguishable populations of hBMSC. Thus, future clinical studies employing hBMSC should attempt to administer the relevant subpopulation of hBMSC dependent on the experimental aim, as a novel approach to improving the clinical efficiency, instead of the current use of heterogeneous hBMSC populations.

## Supplementary Material

List of primers used for real time qPCR.

## Figures and Tables

**Table 1:** Microarray data analysis showing genes related to bone development and osteoblast differentiation upregulated in CL1 versus CL2 cells.

Probe ID	Genbank accession	Gene name	Gene symbol	FC
A_23_P167367	NM_153426	Paired-like homeodomain 2	PITX2	308.84
A_23_P13907	NM_000618	Insulin-like growth factor 1 (somatomedin C)	IGF1	118.00
A_23_P55749	NM_015719	Collagen, type V, alpha 3	COL5A3	75.11
A_23_P94397	NM_005014	Osteomodulin	OMD	56.07
A_24_P128442	NM_152380	T-box 15	TBX15	54.38
A_33_P3708413	NM_003480	Microfibrillar associated protein 5	MFAP5	53.73
A_24_P72064	NM_000163	Growth hormone receptor	GHR	51.47
A_23_P215454	NM_001278939	Elastin	ELN	50.15
A_24_P200854	NM_006735	Homeobox A2	HOXA2	44.99
A_23_P19624	NM_001718	Bone morphogenetic protein 6	BMP6	41.43
A_23_P500501	NM_000142	Fibroblast growth factor receptor 3	FGFR3	33.95
A_23_P154605	NM_018837	Sulfatase 2	SULF2	29.57
A_23_P28815	NM_000782	Cytochrome P450, family 24, subfamily A, polypeptide 1	CYP24A1	22.47
A_23_P210109	NM_019885	Cytochrome P450, family 26, subfamily B, polypeptide 1	CYP26B1	22.42
A_23_P323180	NM_006898	Homeobox D3	HOXD3	21.08
A_32_P405759	NM_152888	Collagen, type XXII, alpha 1	COL22A1	20.36
A_33_P3363799	NM_001242607	Neural cell adhesion molecule 1	NCAM1	17.33
A_33_P3381378	NM_001257096	Paired box 1	PAX1	17.12
A_23_P383009	NM_000599	Insulin-like growth factor binding protein 5	IGFBP5	14.67
A_33_P3382856	NM_133507	Decorin	DCN	14.38
A_23_P10206	NM_005328	Hyaluronan synthase 2	HAS2	14.33
A_24_P77904	NM_018951	Homeobox A10	HOXA10	13.64
A_23_P2814	NM_005905	SMAD family member 9	SMAD9	12.45
A_23_P88404	NM_003239	Transforming growth factor, beta 3	TGFB3	12.11
A_32_P4595	NM_000337	Sarcoglycan, delta (35 kDa dystrophin-associated glycoprotein)	SGCD	8.95
A_23_P162171	NM_006500	Melanoma cell adhesion molecule	MCAM	8.60
A_24_P38276	NM_003505	Frizzled class receptor 1	FZD1	7.81
A_23_P24129	NM_012242	Dickkopf WNT signaling pathway inhibitor 1	DKK1	7.04
A_33_P3264528	NM_005523	Homeobox A11	HOXA11	6.64
A_33_P3220470	NM_005585	SMAD family member 6	SMAD6	6.47
A_23_P23783	NM_000261	Myocilin, trabecular meshwork inducible glucocorticoid response	MYOC	6.41
A_33_P3263432	NM_003637	Integrin, alpha 10	ITGA10	6.35
A_23_P383009	NM_000599	Insulin-like growth factor binding protein 5	IGFBP5	6.18
A_33_P3219090	NM_005542	Insulin induced gene 1	INSIG1	5.78
A_23_P162589	NM_001017535	Vitamin D (1,25-dihydroxyvitamin D3) receptor	VDR	5.68
A_23_P374695	NM_000459	TEK tyrosine kinase, endothelial	TEK	5.65
A_24_P261169	NM_006378	Sema domain, immunoglobulin domain (Ig), transmembrane domain (TM), and short cytoplasmic domain, (semaphorin) 4D	SEMA4D	5.41
A_33_P3297930	NM_005202	Collagen, type VIII, alpha 2	COL8A2	5.138
A_23_P206359	NM_004360	Cadherin 1, type 1, E-cadherin (epithelial)	CDH1	5.12
A_24_P264943	NM_000095	Cartilage oligomeric matrix protein	COMP	5.07
A_33_P3214948	NM_014767	Sparc/osteonectin, cwcv, and kazal-like domains proteoglycan (testican) 2	SPOCK2	4.54
A_24_P55496	NM_053001	Odd-skipped related transcription factor 2	OSR2	4.38
A_24_P354689	NM_004598	Sparc/osteonectin, cwcv, and kazal-like domains proteoglycan (testican) 1	SPOCK1	4.23
A_23_P69030	NM_001850	Collagen, type VIII, alpha 1	COL8A1	3.93
A_23_P128084	NM_002206	Integrin, alpha 7	ITGA7	3.91
A_24_P3249	NM_000965	Retinoic acid receptor, beta	RARB	3.91
A_24_P168574	AJ224867	GNAS complex locus	GNAS	3.83
A_23_P320739	NM_002397	Myocyte enhancer factor 2C	MEF2C	3.74
A_23_P429383	NM_014213	Homeobox D9	HOXD9	3.54
A_23_P42322	NM_080680	Collagen, type XI, alpha 2	COL11A2	3.42
A_23_P160318	NM_001856	Collagen, type XVI, alpha 1	COL16A1	3.36
A_33_P3407013	NM_000600	Interleukin 6	IL6	3.30
A_23_P315364	NM_002089	Chemokine (C-X-C motif) ligand 2	CXCL2	3.29
A_33_P3413168	BC007696	Collagen, type XXVII, alpha 1	COL27A1	3.08
A_23_P43164	NM_015170	Sulfatase 1	SULF1	3.05
A_23_P58676	NM_001204375	Natriuretic peptide receptor 3	NPR3	3.04
A_33_P3290562	NM_000168	GLI family zinc finger 3	GLI3	3.00
A_23_P69497	NM_003278	C-type lectin domain family 3, member B	CLEC3B	2.99
A_24_P353619	NM_000478	Alkaline phosphatase, liver/bone/kidney	ALPL	2.99
A_33_P3305749	NM_000965	Retinoic acid receptor, beta	RARB	2.89
A_23_P307328	NM_007331	Wolf-Hirschhorn syndrome candidate 1	WHSC1	2.77
A_23_P152305	NM_001797	Cadherin 11, type 2, OB-cadherin (osteoblast)	CDH11	2.77
A_23_P216361	NM_021110	Collagen, type XIV, alpha 1	COL14A1	2.62
A_23_P7313	NM_001040058	Secreted phosphoprotein 1	SPP1	2.60
A_24_P267592	NM_015474	SAM domain and HD domain 1	SAMHD1	2.54
A_23_P210482	NM_000022	Adenosine deaminase	ADA	2.52
A_23_P148047	NM_000958	Prostaglandin E receptor 4 (subtype EP4)	PTGER4	2.51
A_23_P345725	NM_014621	Homeobox D4	HOXD4	2.47
A_24_P125283	NM_001015053	Histone deacetylase 5	HDAC5	2.37
A_33_P3231953	NM_004370	Collagen, type XII, alpha 1	COL12A1	2.34
A_24_P298027	NM_004655	Axin 2	AXIN2	2.31
A_24_P336551	NM_199173	Bone gamma-carboxyglutamate (gla) protein	BGLAP	2.29
A_33_P3313825	XM_006713316	Transforming growth factor, beta receptor II (70/80 kDa)	TGFBR2	2.23
A_23_P82990	NM_033014	Osteoglycin	OGN	2.20
A_32_P24585	NM_001017995	SH3 and PX domains 2B	SH3PXD2B	2.20
A_24_P944458	NM_016133	Insulin induced gene 2	INSIG2	2.14
A_23_P99063	NM_002345	Lumican	LUM	2.12
A_32_P5251	NM_001024809	Retinoic acid receptor, alpha	RARA	2.11
A_24_P935491	NM_000090	Collagen, type III, alpha 1	COL3A1	2.10
A_33_P3312104	NM_025099	CTS telomere maintenance complex component 1	CTC1	2.06
A_33_P3321342	NM_016133	Insulin induced gene 2	INSIG2	2.04
A_23_P100486	NM_206824	Vitamin K epoxide reductase complex, subunit 1	VKORC1	2.02
A_23_P53588	NM_030775	Wingless-type MMTV integration site family, member 5B	WNT5B	2.00
A_23_P616356	NM_001291902	Low density lipoprotein receptor-related protein 5	LRP5	2.00

**Table 2:** Microarray data analysis showing genes related immune modulation and immune defense genes upregulated in CL2 versus CL1 cells.

ID	Gene name	Gene symbol	Fold change
A_23_P128094	ATP-binding cassette, subfamily B (MDR/TAP), member 9	ABCB9	2.1
A_32_P156963	Actin, gamma 1	ACTG1	3.0
A_23_P28279	ARP1 actin related protein 1 homolog B, centractin beta (yeast)	ACTR1B	2.0
A_23_P211207	Adenosine deaminase, RNA-specific, B1	ADARB1	3.5
A_23_P381261	Adenylate cyclase 4	ADCY4	5.0
A_23_P169993	Adenylate cyclase 8 (brain)	ADCY8	3.2
A_23_P76823	Adenylosuccinate synthase-like 1	ADSSL1	7.0
A_23_P135486	Alpha hemoglobin stabilizing protein	AHSP	2.6
A_23_P216023	Angiopoietin 1	ANGPT1	7.6
A_23_P94501	Annexin A1	ANXA1	2.6
A_23_P121716	Annexin A3	ANXA3	346.8
A_23_P6398	Adaptor-related protein complex 1, beta 1 subunit	AP1B1	2.2

A_23_P120931	Apolipoprotein B mRNA editing enzyme, catalytic polypeptide-like 3C	APOBEC3C	2.2
A_23_P132316	Apolipoprotein B mRNA editing enzyme, catalytic polypeptide-like 3D	APOBEC3D	2.4
A_23_P357101	Apolipoprotein B mRNA editing enzyme, catalytic polypeptide-like 3F	APOBEC3F	2.3
A_23_P143713	Apolipoprotein B mRNA editing enzyme, catalytic polypeptide-like 3G	APOBEC3G	9.6

A_23_P93988	Rho guanine nucleotide exchange factor (GEF) 5	ARHGEF5	8.7
A_24_P20383	Actin related protein 2/3 complex, subunit 4, 20 kDa	ARPC4	2.2
A_23_P208389	AXL receptor tyrosine kinase	AXL	2.0
A_33_P3279353	Azurocidin 1	AZU1	4.8
A_33_P3262043	BCL2-associated agonist of cell death	BAD	2.3
A_24_P159648	BAI1-associated protein 2	BAIAP2	2.2

A_23_P370682	Basic leucine zipper transcription factor, ATF-like 2	BATF2	22.2
A_23_P160720	Basic leucine zipper transcription factor, ATF-like 3	BATF3	3.0

A_33_P3229272	Breast cancer antiestrogen resistance 1	BCAR1	3.3
A_23_P210886	BCL2-like 1	BCL2L1	3.4
A_23_P98350	Baculoviral IAP repeat containing 3	BIRC3	25.0
A_23_P31725	B lymphoid tyrosine kinase	BLK	7.3
A_33_P3419785	BCL2/adenovirus E1B 19 kDa interacting protein 3	BNIP3	7.3
A_19_P00802936	BRICK1, SCAR/WAVE actin-nucleating complex subunit	BRK1	2.2

A_23_P2431	Complement component 3a receptor 1	C3AR1	2.6
A_23_P97541	Complement component 4 binding protein, alpha	C4BPA	2.6
A_23_P92928	Complement component 6	C6	4.0
A_23_P213857	Complement component 7	C7	2.2

A_33_P3745146	Cell adhesion molecule 1	CADM1	34.3
A_23_P250347	Calcium/calmodulin-dependent protein kinase IV	CAMK4	3.4
A_23_P253791	Cathelicidin antimicrobial peptide	CAMP	3.3
A_23_P82324	Caspase recruitment domain family, member 11	CARD11	7.9
A_23_P500433	Caspase recruitment domain family, member 9	CARD9	2.4
A_23_P202978	Caspase 1, apoptosis-related cysteine peptidase	CASP1	2.4

A_23_P123853	Chemokine (C-C motif) ligand 19	CCL19	2.0
A_23_P17065	Chemokine (C-C motif) ligand 20	CCL20	14.6
A_23_P215484	Chemokine (C-C motif) ligand 26	CCL26	2.8
A_23_P503072	Chemokine (C-C motif) ligand 28	CCL28	4.3
A_33_P3316273	Chemokine (C-C motif) ligand 3	CCL3	2.3
A_23_P152838	Chemokine (C-C motif) ligand 5	CCL5	2.7
A_23_P78037	Chemokine (C-C motif) ligand 7	CCL7	16.0
A_23_P207456	Chemokine (C-C motif) ligand 8	CCL8	2.8

A_23_P361773	Cyclin D3	CCND3	2.3

A_33_P3284508	CD14 molecule	CD14	4.2
A_23_P259863	CD177 molecule	CD177	2.7
A_33_P3381513	CD274 molecule	CD274	10.8
A_23_P15369	CD300 molecule-like family member b	CD300LB	2.0
A_23_P416747	CD3e molecule, epsilon (CD3-TCR complex)	CD3E	2.7
A_24_P188377	CD55 molecule, decay accelerating factor for complement (Cromer blood group)	CD55	5.9

A_23_P300056	Cell division cycle 42	CDC42	4.5
A_32_P148710	Cofilin 1 (nonmuscle)	CFL1	2.8
A_33_P3217584	Cholinergic receptor, nicotinic, alpha 4 (neuronal)	CHRNA4	2.9
A_33_P3415300	Complexin 2	CPLX2	2.7
A_23_P133408	Colony stimulating factor 2 (granulocyte-macrophage)	CSF2	16.5
A_33_P3396139	Cytotoxic T-lymphocyte-associated protein 4	CTLA4	3.0
A_33_P3287631	Cathepsin B	CTSB	2.3
A_33_P3283480	Cathepsin C	CTSC	8.2
A_23_P7144	Chemokine (C-X-C motif) ligand 1 (melanoma growth stimulating activity, alpha)	CXCL1	6.5

A_33_P3712341	Chemokine (C-X-C motif) ligand 12	CXCL12	4.8
A_33_P3351249	Chemokine (C-X-C motif) ligand 16	CXCL16	11.6
A_23_P315364	Chemokine (C-X-C motif) ligand 2	CXCL2	3.3
A_24_P183150	Chemokine (C-X-C motif) ligand 3	CXCL3	2.9
A_23_P155755	Chemokine (C-X-C motif) ligand 6	CXCL6	5.0
A_33_P3214550	Chemokine (C-X-C motif) receptor 2	CXCR2	2.0
A_33_P3389230	Chemokine (C-X-C motif) receptor 3	CXCR3	2.3

**(a):** 

Gene ID	Fold change CL1 versus CL2

FOLR3	28.4721
CCL3L3	17.936
POSTN	15.5924
SERPINB2	−17.2599
IGFBP5	14.6708
CCL3	13.1203
NOV	11.2921
ACTG2	10.4493
CRYAB	10.0678
PSG4	9.68913
RAB3IL1	9.16897
SCIN	9.13702
MYL9	9.12814
TNFRSF11B	8.86049
TAGLN	8.75581
CDH12	8.06682
SHISA2	8.0291
THBS1	7.86854
SPP1	7.64205
LCE2A	7.41042
TMEM98	7.38011
PSG7	7.12183
MYPN	7.01837
FNDC1	6.88102
TNS3	6.72083
ABI3BP	6.67822
LRP3	6.64307
MMP3	6.34715
FAM167A	6.02684
HSPB2	6.01063
ALPL	6.01022
CTSK	5.87356
CXCL12	5.68572
THY1	4.89445
CDH10	4.86105

**(b):** 

Name	*p* value	# molecules
Physiological system development and function		
Organismal development	7.05*E* − 10–1.43*E* − 03	181
Embryonic development	1.00*E* − 09–1.43*E* − 03	154
Organ development	1.00*E* − 09–1.43*E* − 03	145
Skeletal and muscular system development and function	1.00*E* − 09–1.02*E* − 03	123
Tissue development	1.00*E* − 09–1.43*E* − 03	236

**(c):** 

Functions annotation	*p* value	# molecules
Skeletal and muscular system development and function upregulated in CL1 cells		
Size of bone	1.43*E* − 06	24
Differentiation of osteoblasts	3.84*E* − 06	25
Mineralization of bone	4.93*E* − 06	19
Bone mineral density	3.65*E* − 05	19

Skeletal and muscular system development and function upregulated in CL2 cells		
Development of muscle	1.00*E* − 09	44
Proliferation of muscle cells	2.29*E* − 06	35
Remodeling of bone	3.63*E* − 06	21
Resorption of bone	3.94*E* − 06	19

**Table 4:** Microarray data analysis showing genes found to be upregulated during adipogenic differentiation and downregulated after ALP KO.

62 common elements in “AD up” and “ALP down”	Gene name	FC (ALP siRNA versus control siRNA)
APOBEC3G	Apolipoprotein B mRNA editing enzyme, catalytic polypeptide-like 3G	−15.938025
IFI44L	Interferon-induced protein 44-like	−11.520283
PAQR5	Progestin and adipoQ receptor family member V	−6.868241
PNMA2	Paraneoplastic antigen MA2	−5.9695344
DUSP23	Dual specificity phosphatase 23	−5.4786854
CLDN23	Claudin 23	−5.1885047
ANKDD1A	Ankyrin repeat and death domain containing 1A	−5.1646647
IL8	Interleukin 8	−4.887188
LRRC23	Leucine rich repeat containing 23	−4.7611775
IL6	Interleukin 6 (interferon, beta 2)	−4.693139
LIFR	Leukemia inhibitory factor receptor alpha	−4.6540866
PTGFR	Prostaglandin F receptor (FP)	−4.457529
FAM134B	Family with sequence similarity 134, member B	−4.403495
CYFIP2	Cytoplasmic FMR1 interacting protein 2	−4.260462
METTL7A	Methyltransferase-like 7A	−4.0480843
APOBEC3F	Apolipoprotein B mRNA editing enzyme, catalytic polypeptide-like 3F	−3.9502614
CA5B	Carbonic anhydrase VB, mitochondrial	−3.93889
ITGA10	Integrin, alpha 10	−3.9143775
FMO3	Flavin containing monooxygenase 3	−3.852087
IMPA2	Inositol monophosphatase 2 (human)	−3.8374884
CDO1	Cysteine dioxygenase, type I	−3.8181455
CCDC68	Coiled-coil domain containing 68	−3.7292893
CXCL1	Chemokine (C-X-C motif) ligand 1 (melanoma growth stimulating activity, alpha)	−3.5942702
IDO1	Indoleamine 2,3-dioxygenase 1	−3.5803545
KCNIP3	Kv channel interacting protein 3, calsenilin	−3.5442894
FADS1	Fatty acid desaturase 1	−3.2951858
LSR	Lipolysis stimulated lipoprotein receptor	−3.2215986
ITGA7	Integrin, alpha 7	−3.1355932
HLA-DMA	Major histocompatibility complex, class II, DM alpha	−3.1347752
APOBEC3B	Apolipoprotein B mRNA editing enzyme, catalytic polypeptide-like 3B	−3.1074922
BMP4	Bone morphogenetic protein 4	−3.0809238
DMBT1	Deleted in malignant brain tumors 1	−3.0760298
RDH5	Retinol dehydrogenase 5 (11-cis/9-cis)	−3.066812
EPAS1	Endothelial PAS domain protein 1	−3.0615559
CDKN3	Cyclin-dependent kinase inhibitor 3	−3.052319
GPC6	Glypican 6	−3.0460389
CDK4	Cyclin-dependent kinase 4	−2.9808035
FKBP5	FK506 binding protein 5	−2.9360793
PDE1B	Phosphodiesterase 1B, calmodulin-dependent	−2.8863106
JAM2	Junctional adhesion molecule 2	−2.884354
TFPI	Tissue factor pathway inhibitor (lipoprotein-associated coagulation inhibitor)	−2.8578906
NT5M	5′,3′-Nucleotidase, mitochondrial	−2.7555947
NFIA	Nuclear factor I/A	−2.7176137
TSPAN31	Tetraspanin 31	−2.627556
ZNF25	Zinc finger protein 25	−2.6183622
SULF2	Sulfatase 2	−2.5464642
MESP1	Mesoderm posterior 1 homolog (mouse)	−2.525513
BCL2L1	BCL2-like 1	−2.5119667
PLTP	Phospholipid transfer protein	−2.4767148
TIMP4	TIMP metallopeptidase inhibitor 4	−2.465897
CYP27A1	Cytochrome P450, family 27, subfamily A, polypeptide 1	−2.4572072
TTC39B	Tetratricopeptide repeat domain 39B	−2.4439611
IL1R2	Interleukin 1 receptor, type II	−2.427431
FMOD	Fibromodulin	−2.4185398
LDLRAD3	Low density lipoprotein receptor class A domain containing 3	−2.4032724
PISD	Phosphatidylserine decarboxylase	−2.3884957
TMEM100	Transmembrane protein 100	−2.384632
CHST2	Carbohydrate (N-acetylglucosamine-6-O) sulfotransferase 2	−2.3805838
APOBEC3F	Apolipoprotein B mRNA editing enzyme, catalytic polypeptide-like 3F	−2.3759322
SCD	Stearoyl-CoA desaturase (delta-9-desaturase)	−2.3524246
SPAG4	Sperm associated antigen 4	−2.280867
MMD	Monocyte to macrophage differentiation associated human	−2.2055967
ASS1	Argininosuccinate synthase 1	−2.1725202
GK5	Glycerol kinase 5 (putative)	−2.1667244
PDE7B	Phosphodiesterase 7B	−2.166515
MT1X	Metallothionein 1X	−2.161843
ACACB	Acetyl-CoA carboxylase beta	−2.1512873
LEPR	Leptin receptor	−2.148686
HIF1A	Hypoxia inducible factor 1, alpha subunit (basic helix-loop-helix transcription factor)	−2.0954225
HEXDC	Hexosaminidase (glycosyl hydrolase family 20, catalytic domain) containing	−2.094836
SARM1	Sterile alpha and TIR motif containing 1	−2.0797038
BBS1	Bardet-Biedl syndrome 1	−2.0146718
SERPING1	Serpin peptidase inhibitor, clade G (C1 inhibitor), member 1	−2.0102212
FAM162A	Family with sequence similarity 162, member A	−2.005807
TCTN1	Tectonic family member 1	−2.0033443
