# Supplementary material for: Characterization of Cellular and Molecular Heterogeneity of Bone Marrow Stromal Cells
Source: Stem Cells Int. 2016 Aug 16;2016:9378081. doi: 10.1155/2016/9378081 (PMC5004045; doi:10.1155/2016/9378081)
Supplement: Supplementary file 1 — List of primers used for real time qPCR. [file 9378081.f1.zip › 9378081 Supplementary Table 3.docx]

**Supplementary Table 3 – Microarray data analysis showing genes related Immune modulation and Immune defense genes upregulated in CL2 cells vs. CL1 (continuation of table 2).**

| A_33_P3406686 | dynactin 1 | DCTN1 | **2.6** |
| --- | --- | --- | --- |
| A_23_P321501 | dehydrogenase/reductase (SDR family) member 2 | DHRS2 | **4.7** |
| A_24_P408704 | dedicator of cytokinesis 2 | DOCK2 | **4.2** |
| A_23_P139704 | dual specificity phosphatase 6 | DUSP6 | **2.4** |
| A_33_P3258392 | endothelin 1 | EDN1 | **3.5** |
| A_33_P3385002 | ELK1, member of ETS oncogene family | ELK1 | **3.7** |
| A_33_P3232945 | coagulation factor II (thrombin) receptor-like 1 | F2RL1 | **15.9** |
| A_23_P138125 | Fas apoptotic inhibitory molecule 3 | FAIM3 | **2.5** |
| A_23_P250122 | family with sequence similarity 20, member C | FAM20C | **2.4** |
| A_33_P3275741 | ficolin (collagen/fibrinogen domain containing) 3 | FCN3 | **4.3** |
| A_33_P3242136 | fibroblast growth factor 1 (acidic) | FGF1 | **4.0** |
| A_33_P3379886 | fibroblast growth factor 2 (basic) | FGF2 | **3.9** |
| A_33_P3400248 | fibroblast growth factor 20 | FGF20 | **3.7** |
| A_33_P3297907 | fibroblast growth factor 22 | FGF22 | **3.4** |
| A_33_P3348714 | fibroblast growth factor 9 | FGF9 | **5.0** |
| A_23_P372923 | fibroblast growth factor receptor 1 | FGFR1 | **2.0** |
| A_24_P206624 | fibroblast growth factor receptor 2 | FGFR2 | **2.4** |
| A_23_P500501 | fibroblast growth factor receptor 3 | FGFR3 | **34.0** |
| A_24_P334130 | fibronectin 1 | FN1 | **3.3** |
| A_33_P3355503 | forkhead box L1 | FOXL1 | **2.2** |
| A_33_P3318796 | follistatin-like 3 (secreted glycoprotein) | FSTL3 | **3.1** |
| A_23_P255126 | GRB2-associated binding protein 3 | GAB3 | **3.4** |
| A_23_P13899 | glyceraldehyde-3-phosphate dehydrogenase | GAPDH | **3.2** |
| A_23_P146922 | growth arrest-specific 6 | GAS6 | **14.8** |
| A_33_P3550894 | GATA binding protein 2 | GATA2 | **3.0** |
| A_33_P3360341 | GATA binding protein 3 | GATA3 | **61.6** |
| A_23_P74290 | guanylate binding protein 5 | GBP5 | **3.2** |
| A_23_P14564 | G protein-coupled receptor 65 | GPR65 | **2.7** |
| A_23_P130836 | granzyme M (lymphocyte met-ase 1) | GZMM | **2.4** |
| A_23_P373521 | heart and neural crest derivatives expressed 2 | HAND2 | **439.4** |
| A_24_P140608 | heparin-binding EGF-like growth factor | HBEGF | **11.6** |
| A_23_P329924 | hydroxycarboxylic acid receptor 2 | HCAR2 | **3.0** |
| A_24_P365526 | hemopoietic cell kinase | HCK | **6.0** |
| A_23_P73429 | hematopoietic cell-specific Lyn substrate 1 | HCLS1 | **8.0** |
| A_24_P125283 | histone deacetylase 5 | HDAC5 | **2.4** |
| A_23_P404162 | histone deacetylase 9 | HDAC9 | **2.0** |
| A_23_P110196 | HECT and RLD domain containing E3 ubiquitin protein ligase 5 | HERC5 | **2.8** |
| A_23_P6596 | hes family bHLH transcription factor 1 | HES1 | **6.7** |
| A_24_P111996 | hemochromatosis | HFE | **9.9** |
| A_23_P47034 | hematopoietically expressed homeobox | HHEX | **4.8** |
| A_33_P3301040 | HERV-H LTR-associating 2 | HHLA2 | **3.1** |
| A_23_P30913 | major histocompatibility complex, class II, DP alpha 1 | HLA-DPA1 | **3.6** |
| A_23_P258769 | major histocompatibility complex, class II, DP beta 1 | HLA-DPB1 | **5.9** |
| A_33_P3424222 | major histocompatibility complex, class II, DQ beta 1 | HLA-DQB1 | **2.4** |
| A_23_P316511 | homeobox B3 | HOXB3 | **4.1** |
| A_24_P416370 | homeobox B4 | HOXB4 | **3.1** |
| A_23_P153320 | intercellular adhesion molecule 1 | ICAM1 | **7.0** |
| A_23_P45871 | interferon-induced protein 44-like | IFI44L | **2.3** |
| A_23_P68155 | interferon induced with helicase C domain 1 | IFIH1 | **2.9** |
| A_23_P72737 | interferon induced transmembrane protein 1 | IFITM1 | **5.4** |
| A_24_P403459 | interferon, alpha 4 | IFNA4 | **2.3** |
| A_23_P302060 | interferon, epsilon | IFNE | **4.3** |
| A_33_P3243887 | interleukin 11 | IL11 | **14.5** |
| A_23_P91943 | interleukin 12A | IL12A | **3.5** |
| A_23_P399156 | interleukin 12 receptor, beta 1 | IL12RB1 | **2.5** |
| A_23_P29953 | interleukin 15 | IL15 | **5.0** |
| A_23_P61057 | interleukin 16 | IL16 | **2.5** |
| A_23_P332820 | interleukin 17A | IL17A | **3.0** |
| A_33_P3339625 | interleukin 17C | IL17C | **3.2** |
| A_23_P104798 | interleukin 18 | IL18 | **9.3** |
| A_24_P208567 | interleukin 18 receptor 1 | IL18R1 | **2.3** |
| A_23_P72096 | interleukin 1, alpha | IL1A | **2.6** |
| A_23_P79518 | interleukin 1, beta | IL1B | **16.4** |
| A_23_P91850 | interleukin 20 receptor beta | IL20RB | **32.9** |
| A_23_P15146 | interleukin 32 | IL32 | **4.0** |
| A_23_P5654 | interleukin 37 | IL37 | **3.2** |
| A_23_P404494 | interleukin 7 receptor | IL7R | **5.0** |
| A_23_P162300 | interleukin-1 receptor-associated kinase 3 | IRAK3 | **20.8** |
| A_23_P55251 | integrin, alpha 3 (antigen CD49C, alpha 3 subunit of VLA-3 receptor) | ITGA3 | **12.1** |
| A_23_P104199 | integrin, beta 1 (fibronectin receptor, beta polypeptide, antigen CD29 includes MDF2, MSK12) | ITGB1 | **2.8** |
| A_24_P318656 | integrin, beta 3 (platelet glycoprotein IIIa, antigen CD61) | ITGB3 | **3.0** |
| A_23_P329112 | Janus kinase 3 | JAK3 | **2.5** |
| A_23_P502590 | killer cell immunoglobulin-like receptor, two domains, short cytoplasmic tail, 4 | KIR2DS4 | **3.1** |
| A_24_P117147 | killer cell immunoglobulin-like receptor, three domains, long cytoplasmic tail, 1 | KIR3DL1 | **3.4** |
| A_23_P104741 | kin of IRRE like 3 (Drosophila) | KIRREL3 | **14.9** |
| A_24_P133253 | KIT ligand | KITLG | **2.4** |
| A_24_P227211 | kinesin light chain 2 | KLC2 | **2.5** |
| A_23_P63798 | Kruppel-like factor 6 | KLF6 | **2.7** |
| A_23_P204208 | killer cell lectin-like receptor subfamily D, member 1 | KLRD1 | **2.5** |
| A_24_P136402 | lysine (K)-specific methyltransferase 2A | KMT2A | **2.5** |
| A_24_P11506 | kynureninase | KYNU | **16.1** |
| A_19_P00316107 | l(3)mbt-like 1 (Drosophila) | L3MBTL1 | **9.9** |
| A_33_P3355230 | leukocyte-associated immunoglobulin-like receptor 1 | LAIR1 | **2.3** |
| A_23_P29773 | lysosomal-associated membrane protein 3 | LAMP3 | **64.6** |
| A_23_P169437 | lipocalin 2 | LCN2 | **7.7** |
| A_23_P204847 | lymphocyte cytosolic protein 1 (L-plastin) | LCP1 | **68.9** |
| A_24_P20630 | lymphoid enhancer-binding factor 1 | LEF1 | **2.7** |
| A_23_P166459 | lectin, galactoside-binding, soluble, 1 | LGALS1 | **2.9** |
| A_23_P63026 | lectin, galactoside-binding, soluble, 8 | LGALS8 | **18.9** |
| A_24_P122137 | leukemia inhibitory factor | LIF | **3.3** |
| A_23_P380181 | LIM domain only 4 | LMO4 | **2.3** |
| A_23_P207319 | mitogen-activated protein kinase kinase kinase 14 | MAP3K14 | **2.6** |
| A_23_P100704 | mitogen-activated protein kinase 7 | MAPK7 | **2.2** |
| A_23_P201483 | mitogen-activated protein kinase-activated protein kinase 2 | MAPKAPK2 | **2.0** |
| A_24_P41850 | mannan-binding lectin serine peptidase 1 (C4/C2 activating component of Ra-reactive factor) | MASP1 | **8.3** |
| A_23_P317324 | MDS1 and EVI1 complex locus | MECOM | **79.2** |
| A_23_P387471 | MHC class I polypeptide-related sequence B | MICB | **2.5** |
| A_24_P33982 | mast cell immunoglobulin-like receptor 1 | MILR1 | **15.4** |
| A_23_P142310 | MAP kinase interacting serine/threonine kinase 2 | MKNK2 | **3.3** |
| A_23_P1691 | matrix metallopeptidase 1 (interstitial collagenase) | MMP1 | **10.9** |
| A_23_P40174 | matrix metallopeptidase 9 (gelatinase B, 92kDa gelatinase, 92kDa type IV collagenase) | MMP9 | **2.5** |
| A_33_P3311795 | v-myb avian myeloblastosis viral oncogene homolog | MYB | **3.9** |
| A_24_P408424 | myosin, heavy chain 9, non-muscle | MYH9 | **2.5** |
| A_23_P138194 | neutrophil cytosolic factor 2 | NCF2 | **5.2** |
| A_32_P160883 | neural precursor cell expressed, developmentally down-regulated 4, E3 ubiquitin protein ligase | NEDD4 | **3.4** |
| A_23_P386254 | NK3 homeobox 2 | NKX3-2 | **13.7** |
| A_23_P403488 | NLR family, pyrin domain containing 10 | NLRP10 | **14.8** |
| A_24_P244356 | NLR family member X1 | NLRX1 | **12.0** |
| A_23_P213699 | neuregulin 2 | NRG2 | **3.7** |
| A_23_P64828 | 2'-5'-oligoadenylate synthetase 1, 40/46kDa | OAS1 | **3.6** |
| A_24_P124624 | oxidized low density lipoprotein (lectin-like) receptor 1 | OLR1 | **7.2** |
| A_23_P340717 | one cut homeobox 1 | ONECUT1 | **3.7** |
| A_32_P154830 | osteopetrosis associated transmembrane protein 1 | OSTM1 | **3.7** |
| A_24_P319113 | purinergic receptor P2X, ligand-gated ion channel, 7 | P2RX7 | **6.2** |
| A_33_P3213707 | programmed cell death 1 ligand 2 | PDCD1LG2 | **16.5** |
| A_33_P3389649 | phosphodiesterase 4D, cAMP-specific | PDE4D | **4.0** |
| A_24_P339944 | platelet-derived growth factor beta polypeptide | PDGFB | **2.6** |
| A_23_P65532 | pellino E3 ubiquitin protein ligase family member 2 | PELI2 | **11.1** |
| A_24_P63347 | platelet factor 4 variant 1 | PF4V1 | **10.8** |
| A_24_P71244 | phosphatidylinositol-4,5-bisphosphate 3-kinase, catalytic subunit delta | PIK3CD | **3.6** |
| A_33_P3619171 | phorbol-12-myristate-13-acetate-induced protein 1 | PMAIP1 | **4.9** |
| A_23_P334664 | promyelocytic leukemia | PML | **2.4** |
| A_23_P41942 | polymerase (RNA) III (DNA directed) polypeptide G (32kD) | POLR3G | **3.6** |
| A_24_P810290 | phosphatidic acid phosphatase type 2 domain containing 1A | PPAPDC1A | **44.7** |
| A_23_P83599 | protein kinase, cAMP-dependent, regulatory, type I, beta | PRKAR1B | **3.4** |
| A_23_P42975 | protein kinase, cAMP-dependent, regulatory, type II, beta | PRKAR2B | **10.6** |
| A_23_P40096 | protein C (inactivator of coagulation factors Va and VIIIa) | PROC | **5.9** |
| A_23_P310274 | protease, serine, 2 (trypsin 2) | PRSS2 | **22.3** |
| A_23_P135257 | protease, serine, 3 | PRSS3 | **22.6** |
| A_33_P3241984 | protein tyrosine phosphatase, non-receptor type 22 (lymphoid) | PTPN22 | **7.9** |
| A_23_P407614 | PYD (pyrin domain) containing 1 | PYDC1 | **30.9** |
| A_23_P388168 | RAB3B, member RAS oncogene family | RAB3B | **4.8** |
| A_23_P8253 | retinoic acid early transcript 1E | RAET1E | **4.3** |
| A_23_P166051 | RanBP-type and C3HC4-type zinc finger containing 1 | RBCK1 | **4.1** |
| A_24_P10137 | regulator of cell cycle | RGCC | **11.8** |
| A_23_P103310 | S100 calcium binding protein A7 | S100A7 | **2.6** |
| A_23_P404481 | sphingosine-1-phosphate receptor 1 | S1PR1 | **18.4** |
| A_24_P335092 | serum amyloid A1 | SAA1 | **61.2** |
| A_23_P256473 | sema domain, immunoglobulin domain (Ig), short basic domain, secreted, (semaphorin) 3C | SEMA3C | **13.5** |
| A_23_P106389 | semaphorin 7A, GPI membrane anchor (John Milton Hagen blood group) | SEMA7A | **4.7** |
| A_24_P295010 | serpin peptidase inhibitor, clade B (ovalbumin), member 9 | SERPINB9 | **31.1** |
| A_23_P139123 | serpin peptidase inhibitor, clade G (C1 inhibitor), member 1 | SERPING1 | **3.2** |
| A_23_P10121 | secreted frizzled-related protein 1 | SFRP1 | **16.0** |
| A_23_P81103 | secreted frizzled-related protein 2 | SFRP2 | **4.2** |
| A_24_P201739 | SH2B adaptor protein 3 | SH2B3 | **2.3** |
| A_33_P3283619 | SH2 domain containing 1A | SH2D1A | **14.0** |
| A_23_P200443 | SHC (Src homology 2 domain containing) transforming protein 1 | SHC1 | **2.7** |
| A_33_P3368159 | Src-like-adaptor 2 | SLA2 | **3.2** |
| A_23_P158725 | solute carrier family 16 (monocarboxylate transporter), member 3 | SLC16A3 | **15.6** |
| A_33_P3316539 | solute carrier family 7 (cationic amino acid transporter, y+ system), member 2 | SLC7A2 | **37.9** |
| A_24_P335620 | solute carrier family 7 (amino acid transporter light chain, L system), member 5 | SLC7A5 | **3.3** |
| A_33_P3315375 | schlafen family member 11 | SLFN11 | **35.2** |
| A_23_P29939 | synuclein, alpha (non A4 component of amyloid precursor) | SNCA | **18.5** |
| A_33_P3303542 | scavenger receptor cysteine rich domain containing (5 domains) | SSC5D | **7.2** |
| A_33_P3388835 | syntaxin binding protein 2 | STXBP2 | **7.7** |
| A_23_P7582 | transcription factor 7 (T-cell specific, HMG-box) | TCF7 | **3.0** |
| A_24_P402438 | transforming growth factor, beta 2 | TGFB2 | **7.6** |
| A_24_P85775 | thymocyte selection associated family member 2 | THEMIS2 | **8.6** |
| A_33_P3280845 | Thy-1 cell surface antigen | THY1 | **5.2** |
| A_23_P376096 | toll-like receptor adaptor molecule 1 | TICAM1 | **3.1** |
| A_33_P3380807 | toll-like receptor 9 | TLR9 | **3.6** |
| A_23_P24031 | T-cell leukemia homeobox 1 | TLX1 | **4.4** |
| A_23_P126908 | tumor necrosis factor receptor superfamily, member 14 | TNFRSF14 | **3.4** |
| A_33_P3322274 | tumor necrosis factor receptor superfamily, member 1B | TNFRSF1B | **49.0** |
| A_23_P30666 | tumor necrosis factor receptor superfamily, member 21 | TNFRSF21 | **3.8** |
| A_33_P3406623 | tumor necrosis factor (ligand) superfamily, member 12 | TNFSF12 | **7.6** |
| A_23_P152620 | tumor necrosis factor (ligand) superfamily, member 13 | TNFSF13 | **2.9** |
| A_23_P115444 | tumor necrosis factor (ligand) superfamily, member 18 | TNFSF18 | **2.7** |
| A_23_P126836 | tumor necrosis factor (ligand) superfamily, member 4 | TNFSF4 | **47.9** |
| A_23_P30435 | TNFAIP3 interacting protein 1 | TNIP1 | **4.5** |
| A_33_P3405839 | thyroid peroxidase | TPO | **6.4** |
| A_23_P210690 | tribbles pseudokinase 3 | TRIB3 | **4.5** |
| A_23_P216655 | tripartite motif containing 14 | TRIM14 | **55.1** |
| A_33_P3374504 | thioredoxin reductase 2 | TXNRD2 | **3.0** |
| A_33_P3422802 | UL16 binding protein 1 | ULBP1 | **4.8** |
| A_23_P145485 | UL16 binding protein 2 | ULBP2 | **3.1** |
| A_33_P3399571 | vanin 1 | VNN1 | **39.5** |
| A_23_P96331 | Wiskott-Aldrich syndrome | WAS | **3.7** |
| A_24_P557479 | XIAP associated factor 1 | XAF1 | **50.1** |
| A_23_P134419 | zona pellucida glycoprotein 3 (sperm receptor) | ZP3 | **10.5** |
