# Supplementary figures and images for: Characterization of Cellular and Molecular Heterogeneity of Bone Marrow Stromal Cells
Source: Stem Cells Int. 2016 Aug 16;2016:9378081. doi: 10.1155/2016/9378081 (PMC5004045; doi:10.1155/2016/9378081)

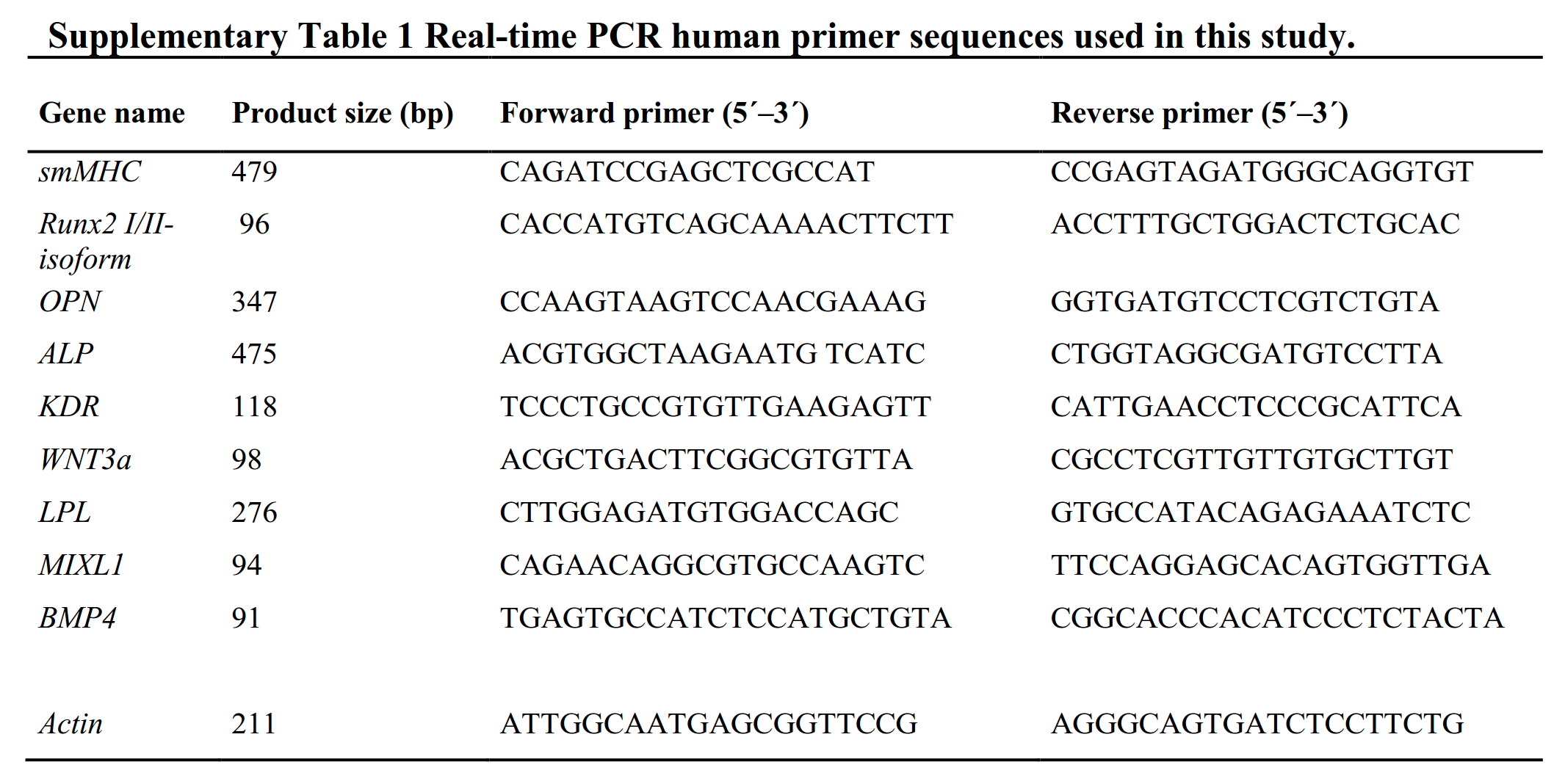

Supplement: Supplementary file 1 — List of primers used for real time qPCR. [file 9378081.f1.zip › 9378081 Suppl Table 1.jpg]

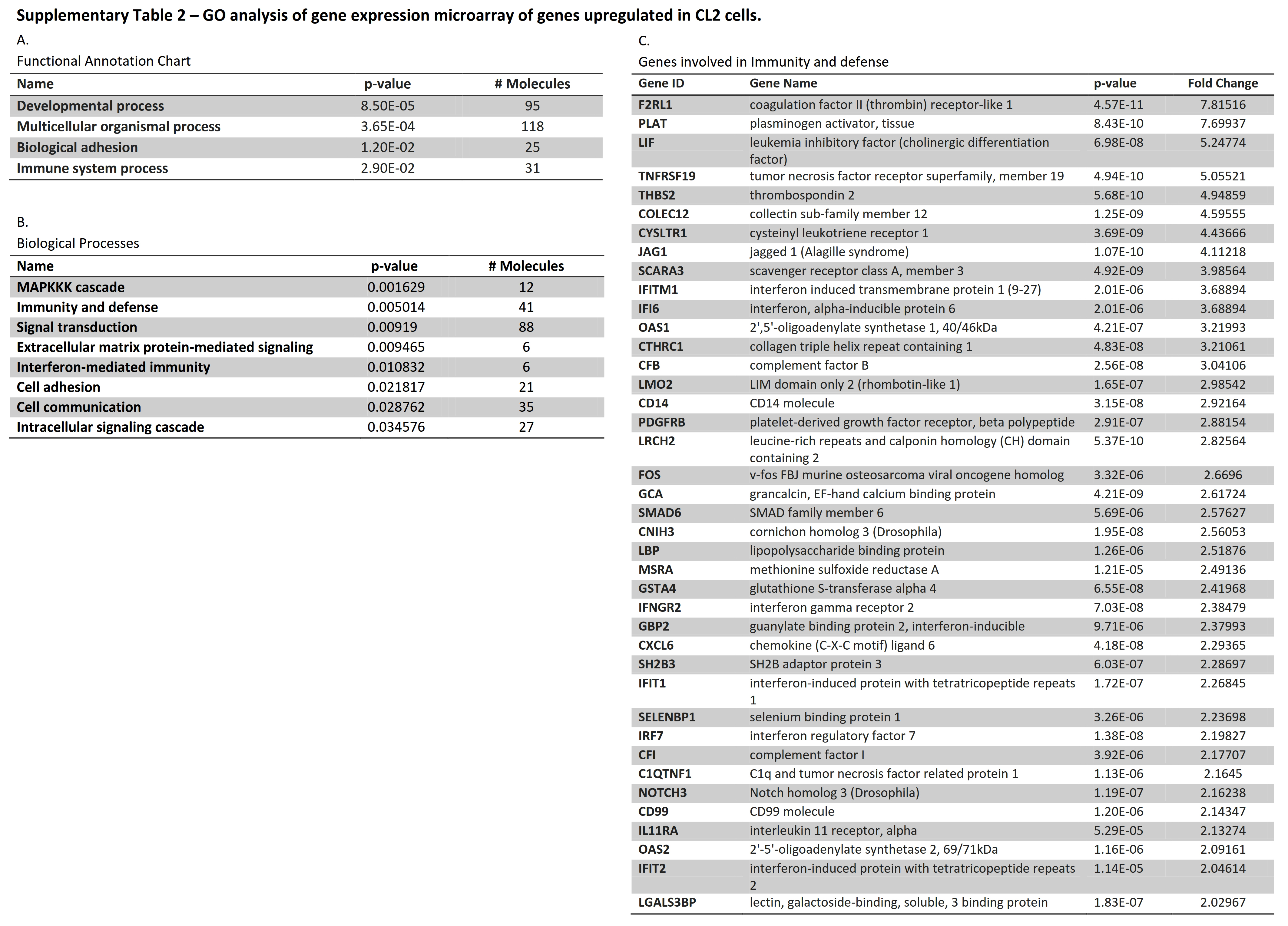

Supplement: Supplementary file 1 — List of primers used for real time qPCR. [file 9378081.f1.zip › 9378081 Suppl Table 2.jpg]

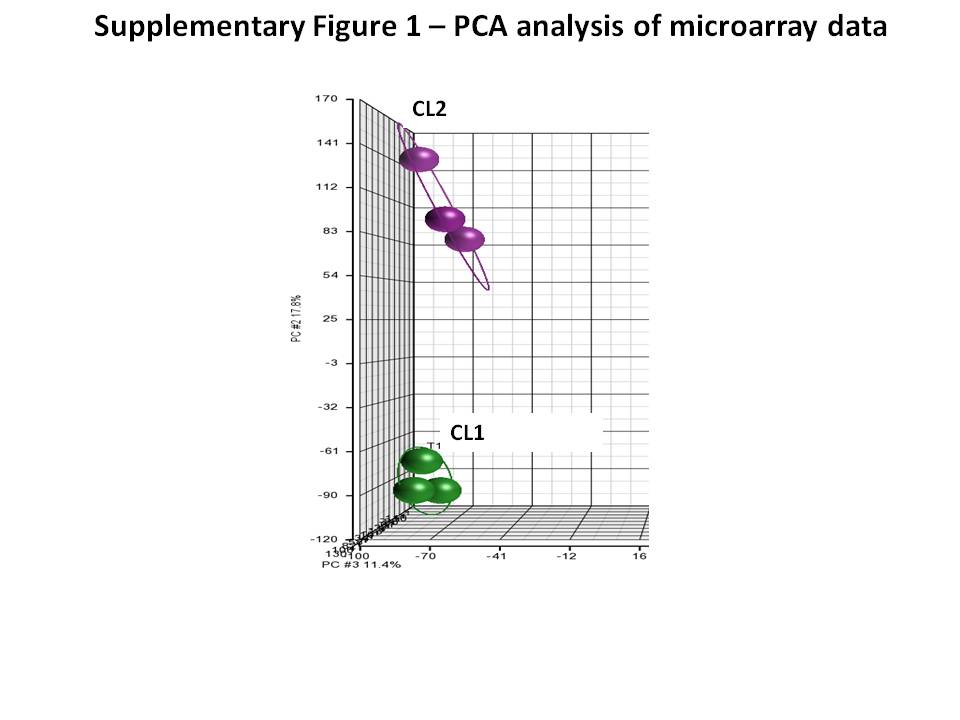

Supplement: Supplementary file 1 — List of primers used for real time qPCR. [file 9378081.f1.zip › 9378081 Supplementary Figure 1.jpg]

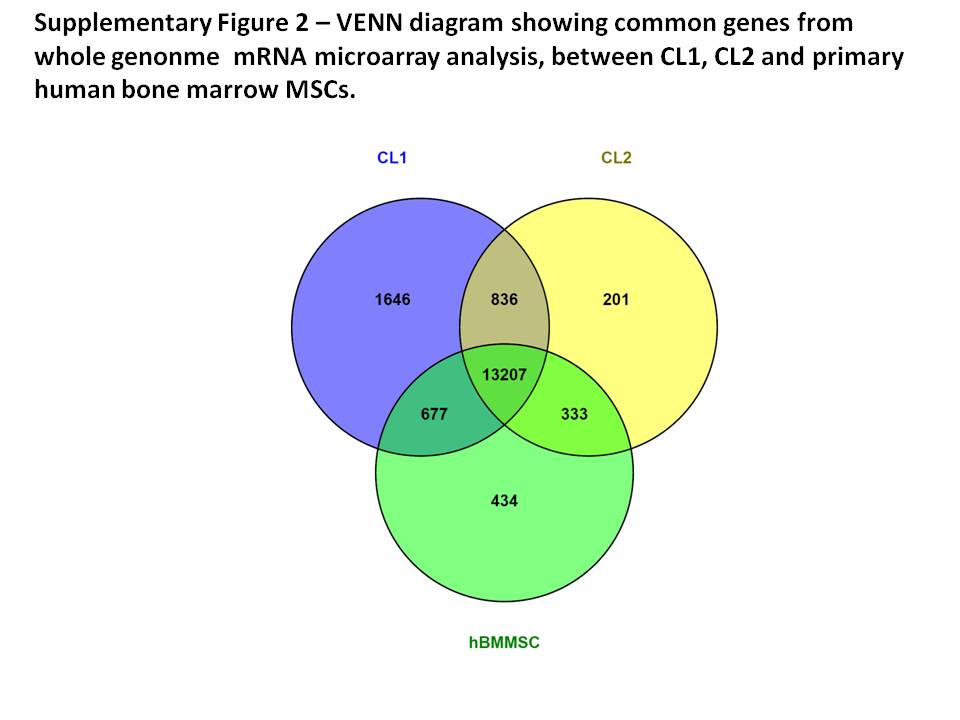

Supplement: Supplementary file 1 — List of primers used for real time qPCR. [file 9378081.f1.zip › 9378081 Supplementary Figure 2.jpg]
